# "The Hospital" Nursing Mirror

**Published:** 1900-06-16

**Authors:** 


"The Hospital" Nursing Mirror.

Being the Nursing Section of "The Hospital."

[Contributions for this Section of "The Hospital" should be addressed to the Editor, The Hospital, 28 & 29, Southampton Street, Strand, London, W.C., and should have the word "Nursing" plainly written in left-hand top corner of the envelope.]

### Notes on News from the Nursing World.

### THE NURSES OF THE IMPERIAL YEOMANRY HOSPITAL.

It will be remembered that Mr. Alfred Fripp, the senior surgeon of the Imperial Yeomanry Hospital, was chaffed and abused alternately for taking what was called by idiots the "ludicrously large staff of 40 nurses" to South Africa. Mr. Fripp, however, has not found the staff too large. On the contrary, he tells us that "he only wishes he had 100." The staff, he says, is "a huge success," and he adds, "They are as comfortable and as safe as in London. They all look very much healthier than they do there, and their work in the wards is, of course, infinitely superior to what any body of orderlies can do." Since Mr. Fripp wrote these comments, the War Office authorities decided to increase the nursing staff of the Imperial Yeomanry Hospital, and the following 10 members of the Army Nursing Service Reserve have left England for the purpose of working at Deelfontein: Misses G. Digwood, K. French, M. A. Macdonald, M. Moore, A. G. Rogers, M. Rogers, J. M. Searle, F. N. Shore, B. J. Talbot, and D. Westbrook. Miss Gertrude Digwood received her training at the Warneford Hospital, Leamington, where she was subsequently ward sister in the men's medical and surgical wards, the women's medical and obstetric wards, and the children's ward. She has since been matron of the Cottage Hospital, Pershore. Miss Mabel Moore was trained at Kent and Canterbury Hospital, and since 1896 has been attached to the Kent and Canterbury Nursing Institute. Miss Ida M. Searle was trained at the East London Children's Hospital and at Guy's, where she was subsequently staff nurse for three years. Since February, 1899, she has been attached to Guy's Hospital Private Nursing Institute. Miss Bessie Isabella Talbot was trained at St. George's Union Infirmary, Fulham, and has since been assistant nurse at the Statutory Hospital, Bath, and charge nurse at the Hospital ships, Long Reach, Dartford.

### NURSING AT MARITZBURG.

An Army Nursing Sister writes us from Maritzburg: "Some of my patients just now are most interesting, not only from the nature of their complaints, but also from their recent experiences. Looking at them one says to oneself, surely 'these have been in great tribulation.' One now down with fever has been a prisoner at Pretoria, but escaped with another man. It was a dreadful time for them both. For eighteen days they lived under the floor, crouching in a space of two-and-a-half feet, and fed only by their fellow prisoners from above. The darkness and want of fresh air must have been awful. Their powers of endurance were tested to the very utmost, but they lived on day by day full of courage, inspired by hope, and confident of victory. When they did get out they had a number of narrow escapes, and this poor fellow is now suffering principally from the effects of all he has gone through. The other day five of us sisters went for a drive in the ambulance waggon, which was drawn by eight mules, the two drivers being black men. We had a real good time, and a first-rate tea in a kind of inn. The landlady was a Jewess, and perhaps a little too talkative. The drive back was the best part, for the sun was setting over the hills."

### A NURSE'S MESSAGE OF CONSOLATION TO A SOLDIER'S WIDOW.

The widow of a private soldier of the 1st Rifle Brigade, who died at Ladysmith of enteric fever, and whose relations reside at Swindon, has received from the sister at the Military Hospital who nursed him the following sympathetic and touching letter:—

"Dear Mrs. Knight,—Until now I have not been able to write to you. I return the letters which were under your husband's pillow. I thought you would not care to have them read by other people. I also send you a lock of his hair which I cut off after his death. He was a patient in my ward, and was not ill very long. The fever was very high when he came in, and it was too much for him. To the last he was a fine, strong, good-looking man. He was so patient and good, and never complained. He was too ill to send any message home, and although he often talked in his delirium we could not make out what he said. Your last letter came when he was too ill to read it. I am so sorry for you in your great sorrow, but it will comfort you a little to know that he had every care and attention, and that everything possible was done to relieve his sufferings. He died very peacefully, and at the last was quite unconscious. May God comfort you and strengthen you to bear this heavy sorrow is the prayer of "Sister Clara.

"Military Hospital, Ladysmith."

### A FALSE ISSUE.

The amateur nurses at the front have found a male champion in the person of Mr. Fletcher Robinson, a newspaper correspondent at Cape Town, who is quite gushing in his description of the manner in which he affirms some of these ladies have ministered to the wants of the soldiers. He relates, in particular, the transformation which, he says, was made in a ward at the Woodstock Military Hospital, by two ladies who obtained permission from the authorities "to provide certain little luxuries." But it has never been alleged that there is any harm done by providing such luxuries as the authorities can see their way to accept. Big basket chairs heaped high with cushions, flowers tastefully arranged in stands, extra pillows, mosquito curtains and screens, are perfectly appropriate gifts, and it would have been churlish to have refused them. Fault is not found with ladies who, in their kindness of heart provide such welcome articles as these, but with the ladies who, posing as nurses, try either to personally force their way into the hospitals, or to smuggle into them things that, instead of being comforts to the patients, are distinctly injurious. We observe, however, that it is urged on behalf of the donors of the little luxuries, that they did not believe that "a badly fitting gown and complete self-abasement before the medical officer, were necessary for successful nursing." Where does the nursing come in? Mr. Robinson's own account shows that it was limited to the decoration of the ward.

### APPEAL OF A NURSE AT THE FRONT.

Mrs. Hildyard, wife of Lieut.-General Hildyard, commanding the Fifth Division in South Africa, has received a letter from a nurse in the Mooi River Hospital, Natal, containing the following pathetic little appeal: "I am often nearly heartbroken when I need things for my patients and cannot get them. We can buy very little out here—horrible tea and biscuits, and only occasionally tobacco. I should be so glad of Benger's food, cocoa, glycerine, biscuits, cigarettes, and condensed milk. These things I need very badly sometimes; also eau de Cologne, which is so refreshing for the enteric cases." Mrs. Hildyard says: "I should be glad to receive contributions for the purchase of the articles mentioned, and propose sending a small parcel every week to the hospital by post." The address of Mrs. Hildyard is 85, Cadogan Gardens, S.W.

### ANOTHER ARMY NURSE MARTYR.

At a meeting of the St. Helens County Borough Council last week, Alderman J. Forster, referring to a resolution passed by the Council two or three months ago, to the effect that all local volunteers who had gone out for service at the Cape should have their names inscribed in tablet form, and placed in position in the Town Hall, suggested that that resolution be improved by the addition of the name of the late Miss Clara Evans, of the Army Nursing Staff, who had died at Bloemfontein. Miss Evans was formerly a resident of St. Helens. The Alderman said he thought the Council would agree that the word "men" might also include women, because Miss Evans and other nurses had gone out to the war with an equally good spirit, and Miss Evans's name might be put on the tablet. They all regretted the loss which the town had suffered by the death of Miss Evans, and he felt that the Council would be pleased to show their sympathy for the family. The Council unanimously agreed to adopt the suggestion.

### A MEMORIAL TO A PIONEER OF NURSING.

We insert with pleasure in another column a letter from Major Ballantine, master of the Manchester Workhouse, in which he mentions a desire on the part of many of the late Miss Hanan's old nurses to give practical expression to the gratitude and affectionate regard for her by contributing towards a suitable memorial of her to be erected in Bristol Cemetery, where she is interred. Major Ballantine anticipates that there are probably other nurses who will be glad to learn of the project, and to send in contributions to him at Crumpsall, Manchester. These should be forwarded not later than the middle of August, and we hope that the memorial to the former Lady Superintendent of Nurses at Crumpsall Infirmary will be of a thoroughly representative character. Major Ballantine merely does simple justice to Miss Hanan in describing her as "one of the pioneers of nursing on the higher plane," and we heartily endorse his tribute to her discernment and devotion.

### MIDWIVES IN THE OTTOMAN EMPIRE.

It will probably surprise many English nurses to hear how particular the Turkish Government is—both in Asia and Europe—about the registration of midwives. "In Beyrout," writes a correspondent, "I recently noticed a sign-board hung high over the front door of a qualified midwife. On it was painted: "Sage femme, diplomée," and the "sign" consisted of a painting of a baby. At Constantinople I came across the same kind of thing, but without the painting of the baby, and the notice was "Sage femme, diplomée, Paris."

### ROMAN CATHOLIC NURSES AND GUY'S HOSPITAL.

Some correspondence which has been published in the *Tablet* shows that certain ecclesiastics of the Roman Catholic Church are, or were, under a curious delusion about the conditions which are imposed upon probationers entering Guy's Hospital. It was gravely stated by a canon of that Church that "a young Catholic girl of seventeen who wants to get into Guy's Hospital, to qualify as a nurse, had seen the matron, who had told her that she must conform in all things to their rules, and attend all the church services." *En passant*, no young girl of seventeen could be admitted on any terms as a probationer at Guy's; but, setting aside the question of age, compulsory attendance is not, as Dr. Perry pointed out in a letter to the secretary of the Catholic Union, a part of the hospital discipline. The Superintendent of Guy's added: "On the contrary, arrangements are made to encourage nurses to attend their own places of worship on Sundays, and at the present time we have upon our nursing staff Roman Catholics, Hebrews, Swedenborgians, and others, who are not members of the Established Church." In enclosing Dr. Perry's letter to the inquiring canon, the Secretary of the Catholic Union expressed his satisfaction, and pointed out that the young lady in question had better make the application six years later.

### PRACTICAL RECOGNITION.

Commenting on the support given by the public to the war funds, a City paper suggests "that something should be done for the great army of women who wage unceasing war with pain and disease, and who are paid on such a scale that adequate provision for advanced years is frequently impossible." Our contemporary having referred in eulogistic terms to the last report of the Royal National Pension Fund for Nurses and the Morgan Benevolent Fund, invites its readers to forward contributions to the secretary. The idea is well meant, but if, instead of sending contributions to the secretary, generous individuals who desire to recognise the devotion of any nurse in South Africa, in whom they are interested, would assist her by making her a policy-holder in the Pension Fund, thus securing her provision in her old age, they would be affording her practical help.

### COLONEL SAUNDERSON, M.P., ON NURSING.

The Portadown District Nursing Society had the good fortune to obtain the services of Colonel Saunderson, M.P., as chairman at the annual meeting the other day. The gallant colonel is one of the most popular members of the House of Commons, and he rarely speaks without saying something that is worth listening to. In commending the objects of the society, he laid great stress on the importance of getting trained nurses to nurse the sick poor. The result at Portadown, he said, had been eminently satisfactory, and the attendance of a skilled nurse in the town had a very large effect in restoring the sick to health. He, however, suggested that, in view of the growth of the population, one nurse was not enough, and he besought his audience to make great efforts to secure the services of another. Meanwhile, the difficulties of the single nurse, who, according to Colonel Saunderson, has more work than one woman can possibly adequately fulfil, are to some extent minimised by the kindness of a lady, who has placed a pony and trap at the disposal of Miss Charlie. The need of a stretcher being mentioned at the meeting, Colonel Saunderson begged that one might be accepted from him as a gift to the society, in memory of the occasion on which he filled the chair.

### THE TRAINING AT WESTMINSTER HOSPITAL.

In the report of a chat with the matron of the Westminster Hospital, which will be found in another part of this paper, Miss Cave made some interesting remarks about the training of the nurses. The four years' system has been enforced for some years; and Miss Cave approves of it, because she thinks that the probationers "should pay back to the hospital something for all the advantages they enjoy during the necessary two years of their training." Miss Cave does not attach supreme importance to the question of time. She thinks that everything depends upon whether a woman has it in her to nurse. Or, to put the case in another way, she says, "At the end of four years one may turn out a machine, but a woman who is suited to the work will be trained in two." There is no dearth of candidates at Westminster Hospital, owing to the four years' training. In fact, Miss Cave states that there are far more applications than can be entertained, even from eligible candidates. Nevertheless, it may be doubted whether the four years' system will become popular.

### THE TRAINING OF MALE NURSES.

Mr. B. Burford Rawlings, secretary and director of the National Hospital for the Paralysed and Epileptic, writes to us as follows: "While acknowledging with satisfaction your appreciative notice of the training of male nurses in this hospital, will you allow me to correct an error which has crept in? The male nurses are not given the "entire charge" of two wards. They work directly under a sister, who is necessarily a fully trained nurse. This, with the present regrettable want of provision for training men in general hospitals, a man cannot be."

### ST. JOHN'S HOSPITAL, LEWISHAM.

The structure of the new building of the St. John's Hospital, Lewisham, is completed, but there is much to be done to the interior. All the pictures have yet to be put in, and the Sisters of St. John the Divine, who are in charge of this centre, are preparing to work hard during the summer in order to get the furnishing ready by the time the walls are thoroughly dry and the new premises habitable. The opening ceremony cannot, therefore, take place for several months. The greater part of the expense of erecting the new hospital, which was badly needed in the neighbourhood, was met by the generosity of one friend; but there is a deficiency in the amount required, and help towards lessening it is gladly received by the Sisters.

### THE NEW SUPERINTENDENT OF THE BEDFORD NURSING INSTITUTE.

Last week we announced the resignation of Miss Rawson, the well-known superintendent of the Bedford Nursing Institute. We now learn, with surprise, that the committee have appointed to the vacancy, at a salary of £40 a year with board and lodging, an untrained person. The lady elected is, it seems, the widow of a clergyman, who was left very badly off, and the motive of the committee in selecting her was kindly. But the movement is none the less retrograde, unless, of course, the new lady superintendent was a trained nurse before she was married.

### HAMPTON NURSING ASSOCIATION.

The annual report of the Hampton Nursing Association is very encouraging, and distinctly confirms the view expressed by Prebendary Ram that the work of the nurse is much appreciated by the people. During last year 212 patients were visited, and 4,380 visits were paid. It is particularly pleasant to notice that the funds received considerable help during the summer from the proceeds of a comic cricket match, and in the winter still more substantial support from the proceeds of a jumble sale. These are practical evidences of the popularity of the association in the place, and the fact that there is a balance of £72 15s. 6d. in hand points to the possibility of employing a second nurse if her services are needed.

### THE NEW NURSES' HOME AT GARTLOCK ASYLUM.

A handsome Home for Nurses has just been opened under the auspices of the Glasgow District Lunacy Board, Gartlock Asylum. It is conveniently placed both with regard to asylum and hospital, and affords accommodation for about 50 nurses. The night nurses are lodged in a part of the home reached by a separate staircase. A bedroom, comfortably furnished, is provided for each nurse, those for the charge nurses being larger than the others. There are four sitting-rooms for day nurses and one for night nurses; on one side of the entrance-hall is a room in which nurses can see their friends, and on the other the matron's room. There are two cloak rooms on the ground floor, and each floor has ample lavatory and bath arrangements. In his speech at the opening ceremony, Dr. Chalmers dwelt strongly on the absolute necessity of providing good accommodation for nurses. He thought it was the duty of the Board to consider that very many of the nurses came from a far distance, "and that it was requisite to provide for them a home—a home away from home, and a good home." His view was that the Board was as much the guardians of the nurses as the guardians of the people who occupied the various wards, and he added that he and his colleagues were determined to do the very best for those taking charge of the inmates. If this spirit could be more generally observed south of the Tweed the difficulties experienced in obtaining nurses would be far less formidable.

### SHORT ITEMS.

Sir Pyers Mostyn has generously undertaken to contribute £80 towards the first year's outlay of a new nursing club in three villages on his estate in Flintshire, and £25 a year annually afterwards. He has also promised to erect a suitable cottage for the accommodation of the nurse, and Sir Pyers and Lady Mostyn have offered to pay the nurse's salary.—At an inquest on Friday last it was stated that one nurse had to look after 33 patients at Bromley Sick Asylum. More nurses are clearly wanted.

### Lectures on Medicine to Nurses.

By H. A. LATIMER, M.D. (Dunelm), M.R.C.S. Eng., L.S.A.Lond.; Consulting Surgeon, Swansea Hospital; Past President of the South Wales and Monmouthshire Branch of Brit. Med. Assoc.; Past President of the Swansea Medical Society; Hon. Life Member of the St. John Ambulance Association, &c.

### STATE MEDICINE.—II.

I AM thankful to say that such rookeries as I described a fortnight ago are being constantly swept away. They are to be met with still, but in greatly diminished numbers, and I doubt not that before long they will be improved off the towns where they have existed. Certain it is that no new dwellings of a similar character will be allowed to be constructed, for the powers that are now held by municipalities and councils are so full and effective that it will be impossible for cramped-up houses of this sort to be erected. One would almost think that the consciences of the holders of property must have been asleep when their owners could have consented to such moral wrong-doing and misuse of God's free gifts of sunlight and fresh air as have obtained in the past. From what I then said I will leave you to imagine what inducements existed for the leading of unhealthy, unhappy, and vicious lives amongst the poor of our overcrowded towns and cities. Were these folk altogether to be blamed if they neglected habits of personal cleanliness when their houses contained only two or three rooms for the use by a whole family, and when such luxuries as bath-rooms were altogether unknown? Could their occupants be expected to stay indoors when they were "cribbed, cabined, and confined" in such surroundings; and if the adults wandered forth into the streets was there much wonder that they should pass into the public-houses which were, and still are, invitingly open to them on all sides? And with a large proportion of their weekly earnings spent in the purchase of drink, is it not easy to see that the proper feeding and clothing of themselves and of their children would be neglected? Indeed, much of the vice and poverty of the past, with much of what, unhappily, still obtains, can be set down to the wretched homes of the people. Now things are steadily improving in this respect, and I can testify from personal experience what changes for the better have taken place within the last two or three decades. The newer streets of towns are well laid out, with proper attention to air space, and many of the houses inhabited by the working-classes are provided with bath-rooms, and all have suitable offices. Go into them, as a medical man and a nurse has to do, and you will be struck with the fact that education is bearing fruit by the evidences afforded by books, musical instruments, and pictures, which you will see about; and reflection will quickly convince you that much of this happy state of affairs is due to the laws which have compelled landlords to build in a healthy manner.

It is time, however, for me to say something about what has been done, and by what means changes have been effected in public health. True progress in such matters dates from 1844, when, after inquiry into the subject matter, the Commission on the Health of Towns issued its report. This report led to the passing by Parliament of the Public Health Act in 1848, and from that time onwards numerous Acts of Parliament have been passed dealing with health affairs as their necessities have presented themselves to the minds of legislators. Perhaps amongst the most beneficial of these enactments have been the ones which have enabled public bodies to borrow monies from the State for the carrying out of public improvements. Heretofore, municipalities had to find the means themselves for work of this character from their own districts; now, on its being shown that the work is really called for properly, loans are issued, and the repayment of the money so obtained is spread over a series of years. It is true that by this measure local communities are becoming heavily indebted to their creditors; but the wealth of the nation is huge, and the security of the rates everywhere is of a first-class character, so that there need be no fear of any default in repayments. Money so obtained has been of incalculable value in enabling the borrowing towns to secure pure water supplies, and to carry out extensive systems of drainage, in both of which matters our country now stands pre-eminent.

Glancing at some of the many enactments which come under the head of the subject I am writing about, we see that unwholesome trades have been placed under Government supervision—this forms a part of the Factory Acts legislation of successive Governments—so that the workmen engaged in them are fenced round with all sorts of protective regulations devised for minimising, or altogether doing away with, the dangerous part of their occupation. Should accidents occur they have to be reported without delay to Government officials; and, should illnesses befall the operatives, the medical men who happen to attend the sufferers are compelled to do the same. The result of all this is that a general improvement in the health of workmen is taking place, especially amongst those who are engaged in dangerous trades, such as in the manufacture of lead, phosphorus, and other poisonous substances.

Food adulteration has also been attended to, and public analysts have been appointed in large numbers, whose duties are to examine specimens of foods which are brought to them from shops in the localities where they hold office. We can readily realise the great good which must follow this health enactment. Take, for instance, the sale of milk deprived of its proper amount of cream and with added water to make up bulk. Here not only is the nutritive value of the food reduced, but a probable source of illness—through a possibly contaminated water supply—is added. In the case of diseased meat, unwholesome fruit, preserved foods, and similar commodities, their condemnation is in the hands of the medical officers of health for the most part, and is generally secured by the sanitary inspectors who work under his direction and now form a well-trained body of men engaged in the various sanitary agencies throughout the country.

You will easily see what beneficial results must follow some of the enactments I will now specify, and the measures which towns are adopting for health purposes. It is no longer allowable for common lodging-houses to be overcrowded; such places are visited by inspectors to see that the regulations under which they are licensed are carried into effect, these regulations strictly limiting the number of their inmates according to the calculated cubic air space of the various rooms. Again, the Notification of Infections Act makes it obligatory for certain scheduled diseases to be reported to the various sanitary authorities which have adopted it, and by this means it is at once possible to trace and to deal with epidemic diseases such as scarlatina and diphtheria, or highly infectious complaints like erysipelas and puerperal fever. And what is still more valuable, many of the sufferers from the former diseases who would, in all probability, if left alone in their own houses with inefficient nursing, have died, are brought under proper treatment at their own homes, or, if they will consent to the change, are removed to hospitals for the treatment of infectious diseases, which are now established in most of the large towns, where they are treated with the happiest effect. The result of these various measures has been that the general death-rate of the country has been lowered from 22·6 per 1,000, which it stood at in 1871, to 17·9 per 1,000 in 1889, and is still being lowered. In the seventeenth century the mortality in London stood at 80 per 1,000; now it only amounts to 20 per 1,000. Such are some of the results of attention to public health.

### The Nurses of Westminster Hospital.

### A CHAT WITH THE MATRON.

By Our Commissioner.

Before I had a chat with the matron of Westminster Hospital she showed me the new rooms which are being provided for the nurses. It is impossible to extend the dimensions of the hospital, and yet increased sleeping accommodation was so urgently needed that something had to be done.

"Eventually," said Miss Cave, when I had seen for myself the great advantages of the alterations, and had looked into two or three of the bright and comfortable wards, "the committee decided to build on the leads on the east side of the hospital, which in olden days were used by the convalescent patients as an airing ground. Now they go to convalescent homes, and the leads are not wanted. We have no home of our own, but twenty beds are assigned to us at the Parkwood Home, Swanley, and the managers of the Marie Celeste Samaritan Fund help us a good deal. The alternative was to put up with extremely inadequate accommodation and with rooms too small to be comfortable."

"The available space appears to me to have been turned to the best possible account. I suppose that even now you have not materially added to the number of rooms?"

### The New Rooms.

"Only to the extent of two. There are thirteen new rooms—six bed-sitting rooms for the sisters, and the remainder for the nurses. The furniture in the sisters' quarters is walnut, and, as you saw, in each room there is a couch bedstead, a corner wardrobe, a bureau combining a writing table and a chest of drawers, a covered-in washstand, a comfortable easy chair, hassocks, an overmantel, a warm rug, and a reading lamp. Except that the furniture of the nurses' bedrooms is in ash there is not much difference. The floors are of block wood, stained and varnished, and the walls are painted primrose and green, which, I think, is a pretty combination and restful to the eye. Then there are servants' cubicles, a bath-room, and a box-room."

"I notice that the electric light is in use?"

"It was put in the hospital last year, and is now used all over the building. The nurses' cubicles on the other side have been enlarged, refurnished, and repainted this year, and the sanitary arrangements improved."

"You have also improved the appearance of the nurses' dining-room."

"That was done last summer when some of the wards were closed. The effect of the white and blue tiles has been to make the room look lighter. The linen-room was repainted at the same time. Of course, the rooms suffer to some extent from absence of light in the basement. But we cannot build without space, and all we can do is to make the most of what we have."

"You have a pleasant sitting-room for the nurses," I said.

"That is the same as it has always been—on the first floor. It has been repainted this year, and a few additions have been made."

### The Training.

"How many nurses live in the hospital?"

"Fifty-two, of whom 12 are sisters. There is a night superintendent, and the remainder are staff nurses. The probationers, of whom I have 26 just now, dine here, but sleep and take the rest of their meals at the Westminster Home for Nurses at Queen Anne's Gate. The matron there is entirely responsible for the private nurses."

"The training here is for four years?"

"Yes; the four years' system has been in force for some time. We consider that the probationers are trained at the end of two years, though we do not grant a certificate until the expiration of four."

"Personally," continued Miss Cave, "I feel that everything depends upon whether a woman has it in her to nurse. At the end of four years one may turn out a machine, but a woman who is suited for the work will be trained in two."

"You see all the candidates?"

"I not only see them all, but I consider that selecting the probationers is one of the greatest responsibilities devolving upon a matron. Candidates come on trial for one, two, or three months. It is easy to tell in a few days that some are not suitable, whilst it takes much more time with others to ascertain their capacities."

"Is there any dearth of candidates owing to the four years' training?"

"None whatever. We have far more applications than we can entertain, even from eligible candidates. That is to say, when we have weeded out those who are too young, too old, too delicate, or in some other way unfit for the work, there are still more than we can find room for. I approve the four years' system because I think that the probationers should pay back to the hospital something for all the advantages they enjoy during the necessary two years of their training."

### Lectures, Classes, &c.

"What are your arrangements as to lectures?"

"Three courses of lectures are given to the probationers, and to supplement the weekly lectures we have classes on the lectures in order to make the nurses thoroughly understand what has been taught them. For many years Dr. Allchin, Senior Physician to the Hospital, has kindly given lectures to the nurses on physiology and medical nursing, and last year the surgical course was given by Mr. Walter Spencer, Surgeon to the Hospital. At the end of each course there is an examination. Last year we were fortunate enough to get Dr. Hayward, of Haydock, to conduct the examination, as an outside Examiner was preferred."

"And this year?"

"This year we call a blank, because so many of the wards being closed we took no new probationers. There are supplemental bandaging, sick cookery, and instrument classes, and I am hoping that the nurses who are fitted for the work will be taught massage. It is important when a nurse trains for a private staff that she should be proficient in massage."

### Hours of Duty.

"When do the probationers come on duty?"

"At 7, and leave off at 8 p.m. The staff nurses come on at the same time, and leave off at 9. Of course there are the intervals for meals. Prayers are said daily in the chapel, once for the day and once for the night nurses. Every member of the staff has two hours off duty, whatever happens, and a day off at the rate of one a month. The sisters have half a day a week off, and the nurses and probationers an evening from 5.30 until 10."

"What about Sunday?"

"Every one of the staff has four hours off on Sunday, and they can all go to their own places of worship, though during the first year I like the probationers to attend chapel. As you know, we are surrounded by churches. A good many nurses enjoy the service at the Guards' Chapel, for which the chaplain gives us tickets. The sisters have a month's holiday, and the nurses and probationers three weeks each. All the nurses sleeping in the hospital can have late passes when they ask for them."

### Recreation Encouraged.

"Then you encourage recreation?"

"I am always glad for any of the staff to go to the theatres, especially if they can go comfortably. We are, therefore, very pleased when tickets are sent us. I think the great object of recreation should be to make the nurse better able to do her work, and more cheerful to her patients. We are rather fortunate in getting permits of admission to the House of Commons. Mr. Erskine, the Sergeant-at-arms, kindly sends them to us, and they are very much appreciated."

"Of course, you have no recreation ground?"

"None whatever; but we have St. James's Park at the back. Our incurable patients go out there. I think cycling benefits the nurses, and sometimes, if we are energetic, we make up parties and run round the park before breakfast."

"Do you possess a library?"

"We have just started one, and have already a fair number of books. We have all the new novels of the day. The nurses pay 2s. a year, which suffices to keep the books in order. The library is open twice a week at stated times. One of the dangers to which a nurse is subjected is that of becoming narrow-minded, owing to the work being so absorbing; and I am sure it is advisable to guard against this by recreation and reading."

PENSIONS.

"Would you like to say anything about pensions?"

"I am very anxious that the committee should see their way to pay half the premiums of the Royal National Pension Fund as at Guy's or the London. I think that the Pension Fund is one of the best things for nurses; it teaches them to be provident."

On this subject Miss Cave—whose professional experience extends over ten years at the London Hospital, where she was sister, and eighteen months at the Metropolitan Hospital, where she was matron, in addition to more than eighteen months at the Westminster—concurred in the opinion that generosity is a characteristic of nurses, becoming almost a fault, though it leans distinctly to virtue's side.

### Which?

SHE talks of "Germs" and "Cases,"
Of "Symptoms," bad and fair;
Of "Disease," which Science traces
To "Microbes" in the air.

She talks as if a patient was
A thing—not man in pain;
A creature that is there because
His cure is Science's gain.

If he gets well—the Doctor's skill
She lauds up to the sky.
He dies—why, then she worries till
She knows the "reason why."

Another, too, is often seen
Passing from bed to bed,
"Germs" are to her a class-room theme;
"Men" lie in "Cases" stead.

Early and late she works for them
(Works with a pleasant smile).
Christ said, "Ye do it unto Me,"
So each Case seems "Worth while."

Suffering and Death she often sees
(For both she does her best),
Gently does all she can to ease,
Leaves in God's hands the rest.

Science is good—in Science's place,
But (give an answer terse)
Which would you like to be, "A Case?"
Or "Patient," nursed by Nurse?

### The Treatment of Burns.

### EXAMINATION QUESTIONS FOR NURSES—RESULT OF MAY COMPETITION.

THE question was as follows: "What would you do in a case of severe burn where the doctor lives several miles away, and probably may not arrive for several hours?"

"Nurse Marie" takes the first prize, and "Workhouse Nurse" the second. The prize winners' answers are short and practical, and show that the writers are alive to the great danger of collapse after severe burns.

"NURSE MARIE'S" ANSWER.

Place patient in bed on a warm blanket, which wrap round the body. If suffering from shock, give at once hot beef-tea, coffee, or brandy, and apply hot water bottles to feet and sides. Cut clothing carefully from affected part or parts, excluding air as much as possible. If large vesicles are formed snip them, and absorb the moisture with cotton-wool or linen, carefully preserving the outer skin as a protection to the inner raw surface. Apply linen or lint soaked in carron oil, olive oil, or lead lotion; but if none of these are at hand dust thickly with fine flour or carbonate of soda. Cover with wool and bandage with flannel binder. Keep patient warm until the doctor arrives.

"WORKHOUSE NURSE'S" ANSWER.

In all cases of severe burn, the attention must first be directed to the alleviation of shock, from which the patient always suffers to a greater or less degree. He must be put to bed in a quiet room, wrapped in a warm blanket, and hot bottles applied to the extremities, whilst stimulants must be administered freely in the form of hot milk, tea, beef tea, &c. If the exhaustion is very severe brandy may be given in small doses. When the patient is somewhat recovered the clothing may be carefully removed and the burnt surface immediately covered with lint or old linen soaked in carron oil, *i.e.*, a mixture of equal parts of linseed oil and lime water. This must be done with as little exposure as possible, as contact with the air greatly aggravates the pain, and may also lead to serious complications. Care must be taken that no blisters are broken in removing the clothing. All portions found to be sticking to the wounds should be allowed to remain, the loose part being simply cut away. If the patient is inclined to sleep he may be encouraged to do so. Symptoms of collapse must be carefully watched for, and the stimulating treatment persevered in.

HONOURABLE MENTION AWARDED TO TWO.

"Nurse Frances" and "Eilrah" receive honourable mention. Speaking generally, the answers this month have been very good, but as usual there has been far too much evidence of slovenliness and carelessness.

EXAMINATIONS CLOSED FOR THE SUMMER.

Before long many will be seeking their summer rest, and we shall, therefore, publish no more questions till the autumn.

### Novelties for Nurses.

### COMFORTABLE CORSETS.

THE Knitted Corset and Clothing Company, of Nottingham, have devised a corset which will be found invaluable for comfort to all women whose occupations call for much bodily movement. Therefore the flexible corset should become most popular amongst nurses. The flexibility is secured by stout ribbon elastic sides to the corset, which do not interfere with support but give perfect flexibility and ease.

### Nursing in America.

By an English Nurse.

I.

Last autumn, various reasons having compelled me to give up work for a time, I thought the opportunity a good one to pay a visit to America, hoping thereby to profit both in health and in pocket. I had no intimate friends there—at least, those I had were settled too far out West to be available—but I had several very kind invitations from people living in the vicinity of Boston, friends of a young American girl whom I had nursed and who had died in a London hotel some years previously. These people had kept "in touch" with me during the intervening years, and as soon as they heard of my intention to come out, at any rate for a few weeks' visit for the winter, if I could find employment, hastened to offer me hospitality. This is not the place to describe the kindness with which I was treated by my "friends over the sea," but my experiences of American nurses and nursing may be of interest.

### Private Nursing.

The first branch of the work with which I became acquainted was private nursing. About the end of October, having made up my mind to remain in Boston for the winter if possible, I set about finding out how they managed things over there—a matter of some difficulty, as no one seemed to know much about it.

"We do not have nurses as you do," one acquaintance remarked, "we prefer to nurse our sick ourselves. You know nurses really are a dreadful nuisance and expense."

However, after interviewing a doctor to whom, among others, I had chanced to be introduced, I began to find out.

### No Advertising Medium.

To commence with, there was absolutely no advertising medium, such as The Hospital, for nurses. There were no "Homes," nor "Associations," which you could join; no means of knowing where there might be a glut of nurses, where a dearth.

"Well, if you really want to try private nursing here," Dr. A. remarked, "though I don't believe you will like it, your best plan is to go down to the 'Directory,' and register, and take your chance, but I am afraid even that is poor. We have two large hospitals here constantly turning out nurses, well known to the doctors in whose wards they have worked. These hang round on leaving hospital thinking their chances of work greatest where they are known, and so Boston, like all the rest of our large cities, is simply glutted with nurses. In fact, except in places down south, where except in the winter the climate is trying, and away out west in the new cities where they have not yet had time to build a hospital of twelve beds which trains and turns out a dozen nurses a year, the whole profession, like my own, is overcrowded. However, it won't hurt to try, and as you have plenty of friends here you may succeed better than one might expect, but I am afraid you are the wrong type and class."

This was not encouraging. However, armed with copies of testimonials I possessed, I proceeded to the Directory.

My first interview was with a lady, to whom I stated frankly just what I wanted—employment for a few months—asking whether she thought it worth my while to register under the circumstances.

She, however, refused to advise one way or the other. "You had better see Dr. B.," she said, "and I will give you his address, and a note."

### "Where did You Graduate?"

To Dr. B.'s house I went next, and, after a short wait, wa called into his office. Doctors don't have rooms, but office in the States. To him I stated my errand.

"'Am—well, I don't know; we have too many nurses already; still, I don't see why you should not do as well as others. Where did you graduate?"

I had not heard the term applied to a nurse's training before, though I was to hear it often enough afterwards, and I supposed I looked surprised. "Where did you train—was it at a recognised training school? Have you got your diploma?"

"St. Thomas' does not give 'diplomas,'" I answered, "but I have got its equivalent, also various testimonials if you care to look at them," handing him some printed copy with which I had provided myself. But he waved them impatiently aside.

### American Ignorance.

"We do not go by testimonials in this country; a nurse stands or falls by her training school."

"St. Thomas' has a training school attached, has it not?" giving me another quick glance over his glasses. "Oh, yes," I answered hastily, feeling somewhat hurt, truth to tell, that everyone did not know the fact. "The Nightingale Training School."

"Ah, yes, of course I had forgotten, and do you know any doctors in this city?"

"I have been introduced to two," I said doubtfully, "but neither of them know anything of my qualifications as a nurse."

"No; well I do not know that it matters much. Take this to Miss —— and pay her the fee. Good morning."

I returned to Miss ——, paid the five dollar fee, and so I was "registered."

### No Nursing Homes.

"And how and where do your nurses live?" I ventured to inquire, when this important ceremony was gone through. "Have you homes or what?"

"No, we have no such things out here; our nurses prefer independence, but if you wish to 'room' I will give you the addresses of several houses where they take nurses only, and where there is a telephone. Let me know which you settle on, and when you are ready for work. Here are some post cards, which you need only fill up; the price is fifteen cents."

"Next week, you think; very well, but don't expect too much. Make friends with the doctors—most of them have their own nurses, and ask for them."

"Is there any additional commission charged for each case?" was my parting question.

"No; each person who sends for a nurse has to pay two dollars; the fee has to be paid by the employer, not the employed."

It sounded uncommonly like a registry office for servants at home, still "different men different minds, different countries different ways."

### My Quarters.

A week later saw me established in the room I had finally selected—or, rather, half a room, for it was shared by another nurse—and for this I was to pay two dollars a week, awaiting further developments. I had made up my mind to give the thing a three weeks' trial, but, truth to tell, was not sanguine. However, my friends had promised to call on me, and I had made engagements enough—conditional, of course, on being still free—to make the time pass pleasantly in any case. There were eight nurses in the house, most of whom had been there for 10, 12, and 15 years; and I do not wonder at it, for Mrs. ——, the owner of the house, was the kindest and best of landladies. But how she took in and nursed a stranger remains to be told.

NURSING IN ENGLAND "WHITE SLAVERY."

These nurses were always most kind to me, and were eager to hear of English nurses and nursing matters, but they belonged to a different type to the people I had up till that time come in contact with, and somehow there were none of them with whom I had any desire to get on to terms of real intimacy. Evidently, things were pretty dull, for I found they had all been waiting at home for several weeks. They seemed to think that not they, but the English private nurse who worked eleven months in the year for £30 or £35 salary, were the ones to be pitied.

"I call it white slavery," one nurse remarked; "why they cannot go on for many years like that. Our plan is much the best. We get a case, are paid 21 or 25 dollars a week, and expect to be at home two or three weeks between each. That gives us time to rest and recuperate, and we go to our next case fresh and well, not wearied out with our last."

The "mealing" at restaurants was a delightful novelty at first, though one tired after a while of never being able to obtain anything to eat without going out for it.

### Appointments.

WHITECHAPEL INFIRMARY.—Miss Eliza Meir Mowat has been appointed Matron. She was trained at St. Thomas's Hospital, and has since been sister at Birmingham Workhouse Infirmary and superintendent at the Workhouse Infirmary, Birkenhead.

KINGSTON UNION WORKHOUSE INFIRMARY.—Miss Ethel Smith has been appointed Charge Nurse. She was trained at the Birmingham Infirmary, and holds a certificate of the L.O.S. Miss Quigley has been appointed Assistant Nurse. She was trained at Birkenhead Union Infirmary.

CITY OF GLASGOW FEVER HOSPITAL, RUCHILL.—Miss Adams, matron of the Middle Wand Isolation Hospital, Motherwell, has been appointed Matron.

### Minor Appointments.

PRESCOT UNION INFIRMARY.—Miss Jessie H. Grimshaw has been appointed Superintendent Nurse. She was trained at Brownlow Hill Infirmary, and has since been home sister at the Nurses' Home, Whiston.

DUMFRIES AND GALLOWAY ROYAL INFIRMARY.—Miss Nancy R. McIntosh has been appointed Staff Nurse. She was trained at Perth Royal Infirmary, and has since been night superintendent at the same institution.

WORKHOUSE INFIRMARY, GRAVELLY HILL.—Miss Gertrude Boylan has been appointed Charge Nurse. She was trained at Chorlton Union Hospital.

### Where to Go.

ROYAL NAVAL AND MILITARY BAZAAR, OLYMPIA, JUNE 19TH, 20TH, 21ST.—There will be 50 stalls representing various ships and regiments belonging to Her Majesty's Navy and Army, decorated with their respective colours, badges and trophies, &c., which will be presided over by the wives of their commanding officers. Concerts, dramatic entertainments, &c., will take place throughout the afternoons and evenings.

THE SCHOOL FOR THE INDIGENT BLIND, ST. GEORGE'S CIRCUS, SOUTHWARK, S.E.—Concert and garden party to the pupils, Wednesday, June 20th, at three p.m.

### OUR CONVALESCENT FUND.

WE have had much pleasure in helping "Nurse K. A." with a grant from the fund. She writes: "I acknowledge receipt of cheque received this morning with my heartfelt gratitude."

### Nursing at Naauwpoort.

A MEMBER of the Army Nursing Reserve Service writes us from Naauwpoort, South Africa, under date of May 17th:—

I am sorry to have to tell you the sad news that one of the Scotch sisters (Boyd) died of dysentery on Tuesday last, May 15th. Her body was taken to the Church Marquee at half-past seven the same evening, draped with the Union Jack and covered with several wreaths. The next morning there was a celebration at half-past six, and at seven a.m. the procession started for the cemetery. It was most impressive altogether. First of all marched representatives of all the Scotch regiments, except the Camerons, in their kilts and khaki coats (they were most anxious to play their bagpipes, but the chaplain wished them not to do so, and it would have made it still sadder had they done so); then followed the gun carriage, drawn by two mules, bearing the coffin, covered with the Union Jack. On either side marched the bearers, who were all non-commissioned officers of the R.A.M.C.; behind the carriage followed the chaplain and three other officers; then came the nursing staff, two and two, and Lady O'Hagan and Miss Thomas, who have charge of the cottage hospital, followed in the rear. Finally came the R.A.M.C., and behind them the civil surgeons and the officers stationed here; in all about one hundred. We walked to the cemetery, a distance of about a mile and a-half. The grave was just as we entered. We have no blank ammunition, so firing is not now allowed, but, instead, the last post ended the service. This was very trying, as it is so shrill. After the service the procession broke up, and we all walked quietly home. Everything was most beautifully arranged and carried out, and the service was very impressive. The Scotch sisters left next day for Norval's Point. We have three sisters down with enteric. I hope others will soon be sent to take their place, as we already have quite as much work as we can do, and more than we can do properly. The latest report is that this is to become a base hospital of 1,500 beds.

### To Nurses.

WE invite contributions from any of our readers, and shall be glad to pay for "Notes on News from the Nursing World," or for articles describing nursing experiences, or dealing with any nursing question from an original point of view. The minimum payment for contributions is 5s., but we welcome interesting contributions of a column, or a page, in length. It may be added that notices of entertainments, presentations, and deaths are not paid for, but, of course, we are always glad to receive them. All rejected manuscripts are returned in due course, and all payments for manuscripts used are made as early as possible at the beginning of each quarter.

### TRAVEL NOTES AND QUERIES.

RULES IN REGARD TO CORRESPONDENCE FOR THIS SECTION.—All questioners must use a pseudonym for publication, but the communication must also bear the writer's own name and address as well, which will be regarded as confidential. All such communications to be addressed "Travel Editor, 'Nursing Mirror,' 28, Southampton Street, Strand." No charge will be made for inserting and answering questions in the inquiry column, and all will be answered in rotation as space permits. If an answer by letter is required, a stamped and addressed envelope must be enclosed, together with 2s. 6d., which fee will be devoted to the objects of the "Hospital Convalescent Fund." Any inquiries reaching the office after Monday cannot be answered in "The Mirror" of the current week.

SCHINZNACH, SWITZERLAND (S. B.)—Expense of journey about £5 5s. second-class return. Cheapest route by Newhaven, Dieppe, and Berne. It is at the foot of the Wülpelsberg. The climate is dry, and is not subject to rapid changes. Not placed very high—1,100 ft. above sea level. The waters taken internally and externally are useful in various skin diseases; also for asthma and rheumatism. Three doctors resident at Schinznach direct the treatment. There is no cure tax, and the charges are moderate.

BRUGES OR BRITTANY (Nurse Sprague).—Why not attend to rules, and send pseudonym? You would find Bruges hot in July, I think, not that the temperature is high, but because Bruges is a closely-built town. It is full of canals, which sometimes smell a good deal in hot weather. It would not be so bracing as Sussex decidedly. Brittany would be more suitable. I do not much care for the Brittany convents. Why not try Paramé, close to Dinard, just across the bay; very bracing; not so expensive as Dinard, but close enough to benefit by its advantages. Will send you addresses of hotels if you wish.

### Echoes from the Outside World.

### AN OPEN LETTER TO A HOSPITAL NURSE.

The sudden, very sudden, advent of summer has made one strangely averse to theatre going, and tickets which would have been hailed with glee last week are at a discount. An American girl whom I met on Monday asked my advice as to where to go, and inquired, in the most casual way, which of our theatres had the best "roof garden"? In my ignorance I stared at her in amazement, a sentiment which was reflected on her face when I told her that London did not possess such a thing. It appears that New York amusement seekers so resent being "broiled" in a theatre in the dog days that the managers realise the uselessness of expecting an audience to undergo the torture, and those houses which do not provide a "roof garden" shut up in summer. I wonder which will be the first enterprising manager to give us this luxury in the biggest capital in the world? Meanwhile, if any of you get the chance of seeing Mr. Ben Greet's series of open-air plays, to be held in the Botanical Gardens, be sure you seize it. It is a most delightful and refreshing performance, and the *entourage* of Regent's Park at night should be most fascinating. The series began on Wednesday, and will end on July 3rd.

I am glad to hear that the clergy in many parts are making a stand against the practice of throwing confetti in church. One would have imagined, in the simplicity of one's heart, that such a protest was unnecessary—that all who valued the blessing of the Church upon their union would naturally respect the sanctity of the building in which the ceremony is performed. But such is not by any means the case. In many places, as soon as the ceremony is over and the clergyman has returned to the vestry, the guests begin throwing the little pieces of paper at each other till the scene is much more suggestive of a carnival or a fair than a religious ceremony. It is difficult to know how to stop the nuisance, because it is obviously impossible to search the guests before they enter the doors; and to appeal to the sense of reverence in a church confetti-thrower would be equally absurd. I wonder if it would be possible to bring such disorderly conduct under the head of brawling in church, because that, I believe, is an offence for which you can be taken into custody. A few bridesmaids and groomsmen in durance vile for a short time would do much to render the practice of throwing about handfuls of confetti unpopular. Indeed, its disappearance from all galas and festivals would be an advantage. At a carnival held on behalf of the Bolingbroke Hospital last week I got such a dose thrown into my eyes once that I could see nothing for the next few minutes. But that sort of incident is, I suppose, to the thrower, merely an unimportant detail.

For the past few days the papers seem to have been full of wars and rumours of wars. The news from South Africa is on the whole satisfactory. Unfortunately the Boers captured the Sherwood Foresters, numbering about 600 officers and men. Against this mishap we must set the surrender of 1,500 Boers in the Orange River Colony, the release of nearly 4,000 English prisoners at Pretoria, and a brilliant victory by Lord Methuen over General de Wet. Lord Airlie is among the killed. Notwithstanding some alarmist assertions that food at Kumasi fort is extremely scarce, that the place contains two hundred wounded men, and that the town has been unable to send any message through for a month, the opinion of those who ought to know is, that it will not be necessary to send further troops to Ashanti to quell the rebellion, as the 2,300 men now under the command of Colonel Willcocks should prove amply sufficient for the purpose. It will be a relief to us all when we hear that the trouble has been quashed. There seems also likely to be mischief between Korea and Japan, but that does not affect us in the same way as the tidings from China. At first it seemed that the disturbance was only temporary misbehaviour on the part of the "Boxers," but from later information it is evident that the Dowager Empress—who always seems to be at the bottom of our Chinese difficulties—is encouraging the revolt. In response to foreign pressure an edict was issued by the Chinese Government, which was supposed to condemn the rising, but instead of blame it has dealt out praise, and the censure has been given, not to the agitators, but to the troops who attacked them. The Ministers are able to do but little personally in the matter, because it appears that none save the German Minister understand Chinese, though one would have imagined that the first business of a delegate to a foreign country was to master the language. Therefore, the drawing up of all notifications has to be left to Chinese officials, who do not scruple to write just the opposite of what they were told to. Two missionaries, Mr. Norman and Mr. Robinson, were hacked to pieces in circumstances of revolting barbarity, and, according to an American telegram, hundreds of native Christians have been massacred by the rebels. The suggestion that the only way to secure a return to peaceful conditions in China would be to seize the person of the Empress—who is strongly anti-foreign in her predilections—and keep her in detention, Formosa being suggested as a convenient spot for her future residence, is, I fear, too simple to be practicable.

Have you read any of the evidence given in the case of the lady calling herself Countess à Court? It is all highly dramatic, and suggests a penny novelette of the most thrilling and improbable character. Had not the lady been sufficiently ill-advised to bring an action for slander against her late employers because they dismissed her upon discovering that, as they allege, though she was engaged by them as a highly-accomplished governess, she had acted as parlourmaid in the neighbourhood under the name of Susan Adams, all the details of her career would probably never have come to light. It is said that "one man in his time plays many parts." Most assuredly one woman sometimes does, if all the evidence given in this instance should prove to refer to the same person. A foreign governess asserts that she handed several German certificates to the prisoner when she applied to her for a situation. These were never returned, but were produced at the Court by the "Countess" as her own. In her capacity as governess this individual spent about ten days with some county people in the Midlands who are friends of a mutual acquaintance. She was very highly recommended, and her position was that of finishing governess to two girls. The latter soon discovered that her capacity to "finish" them was limited, and they were considerably amused at the numerous questions she asked them as to the amount and value of their mother's jewellery. Evidently she was not afraid of her questions being deemed too inquisitive, as one day at lunch she inquired of her hostess "whether the pearls round her neck were real." Miss à Court left of her own accord. She had, she said, received an offer of marriage from a gentleman in South Africa, who had sent her a most valuable engagement ring, and £60 to pay her passage out, and she would be glad if her employer would dispense with her services. Her employer consented, and the girls afterwards received a letter from their late governess from Las Palmas on her outward journey. It was apparently some time prior to this that she had been for two days head parlourmaid to a lady of title, arriving in a black silk dress and a white overcoat, and having as luggage only a dress basket and a bundle of golf sticks! A parlourmaid with a bicycle as part of her luggage is by no means unusual, but the golf-playing maid suggests a new development.

### The Properties of Leben.

By a Nurse.

In a recent article upon "Nursing in the Holy Land," mention was made of the good dietetic properties of leben, and as few people, unless they have travelled in Syria, are likely to know much about this excellent food for invalids, especially suitable in cases of exhausting fevers, some remarks on the subject may be of interest to our readers.

Leben is essentially a Syrian food, and one, moreover, of great antiquity. In Judges iv. 19, it is stated that Jael, the wife of Heber the Kenite, gave Sisera milk to drink out of a bottle. Now, in the Arabic Bible the word "milk" is translated, and far more faithfully, as "leben," and to one acquainted with the customs of these "dwellers in tents" this passage brings before their mind the familiar picture of a Bedouin tent and a large rough leather bag made of goat's skin hanging at the entrance filled with "leben."

### The Mode of Preparation.

To describe this delicious drink as sour milk, or as similar to curds and whey, would be distinctly misleading. The following is the mode of preparation: The fresh milk is taken warm from the cow or goat and poured into "tungaras"—round, shallow vessels of earthenware or brass. There is then put into it a little piece of "roubie" or dried portion of stale "leben." The vessels are next put on one side, and if the weather is cold they are covered with a cloth in order that the milk may retain a certain amount of warmth. The "tungaras" are then left undisturbed till the morning. By that time the coagulating bacteria contained in the "roubie" has done its work and the milk has undergone a change, and has the consistency of curds and whey. In appearance it is peculiarly white, as its name implies. Leben and Lebanon, the White Mountain, from the snow covering its higher ridges in winter, are both taken from the same root. It has a strong acid, not sour, taste, and a slightly acid, pungent smell. After a long, hot ride nothing is more refreshing than a bowl of "leben." It quenches the most tormenting thirst as no water can, and at the same time restores the exhausted energies. It also has a slightly soporific effect. I have been told by Syrian women that, failing to obtain a portion of "roubie" to turn the milk, an almost unheard-of thing, they put fresh cut twigs of the fig tree into it, and they answer the purpose. What peculiar bacteria is developed in the milk when the fig tree twigs are used as a coagulent is still a point of scientific discussion.

### A Daily Article of Food.

Leben is consumed as a daily article of food by all classes of Syrians. It is most generally eaten with the thin brown bread of the country, but it enters largely into the native cookery. Sometimes with rice, at others with meat, also mixed with olive oil, as a dressing to the nameless roots, gathered in the wilderness, that go by the general name of salad.

### Its Medical Value.

Regarded from a medical point of view, leben is of immense value. It has all the nourishing properties of milk, while it is lighter and more easy of digestion. In cases of typhoid, dysentery, and malaria one cannot speak too highly of its virtues. Milk, as we all know, is apt to pall sadly on the fever-stricken patient, and its sickly taste becomes detestable in a hot climate, unless it is iced, and ice is almost impossible to obtain for love or money in the height of a Syrian summer. But leben, with its clean, cool, acid taste is most welcome. Many a patient I have fed on it, and when, unfortunately, a patient myself with dysentery, scarcely anything but leben passed my lips for a fortnight.

### Outward Application.

It also has its virtues as an outward application, as I can testify from personal experience. On coming off a long, hot journey, with one's face painfully burnt by the sun, leben is excellent if applied at once and left on till next day. It will then be found to have almost entirely drawn the burn out of the skin. I was once praising it to a native doctor for these qualities, and he forthwith declared he would recommend it to a patient of his, a Syrian dame of high degree, who was continually on the look-out for some new aid to revive her fading charms. In an unlucky hour for him he did so, with the result that in a few days he came to inform me that I had caused him, temporarily at least, to lose a wealthy patient. On my expressing surprise, he explained that the Syrian beauty, on hearing of the new aid to a good complexion, immediately applied it plentifully, forgetting that her face was already covered with a cheap French cosmetic, with the awful result that her skin turned nearly black. The acid of the "leben" was evidently not compatible, as the Pharmacopœia would say, with something in the cosmetic.

### A Week in Paris.

By a Nurse.

For the benefit of other nurses or individuals who want to visit Paris during the Exhibition in a comfortable, and at the same time inexpensive way, I will describe my method.

I made the sixth of a party, and we went to Paris entirely on our own account, without the aid of any tourist company. We took third-class return tickets, 26s. each, viâ Newhaven and Dieppe, leaving London Bridge Station by the 8.50 p.m. train on Friday, May 18th, and arriving in Paris at 7.30 a.m. on Saturday. We paid a few shillings extra for a first-class passage going, but not coming home. This may be done on the steamer.

Before going we had written and secured rooms at Nos. 10 and 20, Rue Clairant, Batignolles, Paris, and I cannot do better than pass on these addresses, as we were so comfortable. The charge is very moderate indeed, being seven francs per day inclusive. It covers a good bedroom and three substantial meals, breakfast, lunch, and dinner. It is wise to be provided with a small stove and a little tea, as the latter is a big item in a week's expenses, and these articles take up very little room in one's bag. If each person can manage with one hand-bag it will be a great convenience. We only took one each, and had no trouble with porters or with tips, which usually mount up tremendously by the time the trip is over.

We planned something for each of the seven days in Paris, as we did not go merely to see the Exhibition, but as many places of interest as we could crowd in in the time. We spent two whole days in the Exhibition, and gave up the rest of the time to visiting Versailles, the Bois de Boulogne, trips up and down the Seine, Notre Dame, the tomb of Napoleon, and as much time as we could put in at the Louvre. We mounted to the top of the Arc de Triomphe, and got a magnificent view of Paris.

I am not going to describe the Exhibition. Suffice it to say we were quite over-awed at its magnitude and splendour, and the impression everything there made on our minds will never be effaced.

There is one thing I must point out about the entrance fee to the Exhibition, namely, that in the daytime the charge is fifty centimes. After six p.m. each person must be possessed of two of these tickets, paying half a franc for each. Many English people have been fleeced over this arrangement, as the tickets have one franc printed on them, whereas they only cost half a franc. The point is to have two tickets and pay one franc.

We left Paris at ten minutes to nine p.m. on Friday, and reached London at eight a.m. on Saturday after a very pleasant and exceedingly full week of sight-seeing, the whole trip costing five and a-half guineas, including a guide at Versailles and all minor expenses.

### Across the Seas.

### NURSING AT THE LEADING HOSPITAL IN CEYLON.

By a Correspondent.

In Ceylon the health of the people is entirely in Government hands. Hospitals have been established throughout the island to correct the effects of "devil dance cures," and to rescue the natives from the hands of vedaralas or quacks, whose treatment is by terrorism or demonology. Official distributions of quinine are made periodically to the natives in fever districts, and a resident dispenser on every tea estate prescribes and dispenses medicines to the coolies. Government medical officers go the rounds of outlying districts where no doctors exist. Often in their peripatetic prescribing tours they hold an out-patient department under the shade of a big banyan tree. In many villages there is no building large enough to accommodate the natives who flock in such numbers to consult the "master doctor."

Government has established a convalescent gaol at Hambantota, where sick prisoners from Colombo are sent for the benefit of a term of "up-country air." Moorish vaccinators are provided for the Moormen or Mohammedans, while women vaccinators meet the prejudices of Mohammedan women, who are not allowed to receive the lymph at any but feminine hands. The Civil General in Colombo is the leading hospital of the island. It covers 14 acres of ground, is situated in delightful cocoa-nut and banana plantations, and contains 356 beds. It is thoroughly up to date in equipment, contains an admirable operating theatre, furnished by Arnold, of London, with sterilisers and all aseptic requirements, while a bacteriological department has lately been added and endowed by Mr. de Soysa, a millionaire burgher.

### The Nursing.

In fact, everything is up to date, except the nursing. So far, all attempts to establish an efficient training school at this hospital have come to nought. Several reasons underlie the failure of this very important department, which at present is in the hands of Franciscan Nursing Sisters. The Ceylon Government has not sufficiently recognised the value of skilled nursing, and so economises in salaries that hospital posts in Ceylon are not professionally attractive. The nursing sisters attached to the Civil General Hospital receive a salary of £4 a month and quarters. Out of this sum they have to provide their own food, attendance, and uniform. Religious sisters, under a vow of poverty, receiving no holidays and needing no provision for old age, can accept these terms. To the lay nurse such pay would not constitute a living wage. The sisters of the Civil General are not trained nurses. Their rules forbid them to perform many duties for the sick which are essential to good nursing. These white-robed attendants are most kind and attentive to the sick. But it is the old story of the inefficient nursing of R.C. Sisterhoods.

### The Language Difficulty.

Most of the sisters of this hospital are French and Belgian. Nearly all of them have learned to speak the native languages with fluency, and this is one special reason why they are not replaced by English-trained nurses. Some members of the Ceylon Government, who are anxious that an efficient training school should exist, complain that English nurses will not learn the native tongues. Medical men throughout the East aver that this fact proves a great stumbling-block to nursing progress in Ceylon and India. All the medical officers appointed in the East are required to make themselves proficient in the tongues "understanded of the people." The nurses should do the same. The first duty the R.C. sisters take on themselves is to study the vernacular. The ability to talk to the patients in their own tongue at once establishes sympathy and confidence.

### The Ordinary Wards.

Some of the wards have cement floors, but these are gradually being replaced by wooden ones. The hospital is on the pavilion plan, and the verandahs and grounds, with their gorgeous tropical flowers and shrubs, afford pleasant airing courts for convalescents. A passenger pavilion is set apart for strangers falling sick by the way. Travellers taken ill in Colombo, or passengers from the myriad steamers calling at this port, are glad to find such pleasant quarters in these wards. Many persons, too ill to be nursed with comfort on board ship, have cause to be thankful to the Ceylon Government for this thoughtful provision. Serious dysentery and enteric cases are not suited to steamers. In these bright, cheerful private rooms the patient may, if he like, engage his own nurse. The moderate sum of five rupees daily (10 shillings according to local spending value, 6s. 8d. by English reckoning) covers the cost of doctor, food, medicine, and attendance.

### The Children's Section.

It was noticeable in the children's section that the mothers and small brothers and sisters of nearly all the patients were also admitted to the wards. This is a native custom. The mothers thoroughly enjoy the change and rest, combined with free board and lodging. There is plenty of society for them among the Cingalese mothers of other patients. They sleep on mats on the ward floor, get in everybody's way, and enjoy themselves amazingly. One thousand cubic feet of air is allowed to each patient. By the time hordes of affectionate relatives are added to the ward the cubic ideal is not reached.

### A Breach of Tradition.

The doctor in charge, who kindly acted as personal conductor, remarked that most of the patients allow quack or magic medicine men to have a first try at their cases before presenting themselves at the hospital. He also mentioned the curious fact that native women treated for anæmia are disgusted when they lose their "fair skins." It would take a native to notice the difference, but the least lightening of the colouring matter of the skin is accounted a great beauty. A few Mohammedan women have begun to assert their right to skilled medical assistance—rendered even in the masculine gender—and have found their way to the hospital. But this is a great breach of tradition, and the neighbouring Lady Havelock Hospital provides for such cases.

### The Native Patients.

Separate wards are set apart for ulcers and diarrhœa. Many of the patients in the latter wards were picked up unconscious in the roads. Diarrhœa is very prevalent among the Cingalese, and a bad case is often taken for cholera by friends and neighbours, and consequently shunned and neglected. Natives used to object to going to the hospital in the fear of being "experimented on." Custom has taught them its value, and now they attend in such shoals and for such slight ailments, that their numbers are sometimes embarrassing to the medical staff. The medical staff consists chiefly of burghers, *i.e.*, the descendants of the Dutch colonists of Ceylon. Many of the burghers have intermarried with the Cingalese, so that the burgher of to-day is very much like the Eurasian of India. The medical staff of the Civil General at Colombo is highly efficient—many of the doctors hold high English qualifications. It is greatly to be desired that a modern training school for nurses should be added to this excellent institution, since such admirable material is ready to hand.

### A Book and its Story.

### ROMANTIC REMINISCENCES.

No recent historical reminiscences are more interesting and romantic than those of the Baroness Cecile de Courtot, lady-in-waiting to the Princess de Lamballe,* whose life-history contained in the series of letters preserved in an old dower chest, and discovered nearly a century later by the author, Moritz von Kaisenberg, have been ably translated by Miss Jessie Haynes for English readers. The letters are addressed to Herr von Kaisenberg's great grandmother, which, supplemented by extracts from her diary, help to make clear the occurrences written of, and to bring forcibly before the reader tragic scenes in the Reign of Terror. There is such a strong human interest running through the narrative, so vivid and fascinating a personality suggested by these letters that, although written a hundred years since, they are as fresh and delightful, with the charm of simple sincerity, as if written to-day. The reader cannot fail to be thrilled with this touching record of a faithful, loyal soul, who "suffered and was strong."

Frau von Alsvenleben, to whom the letters were written, must have been a woman of unusual gifts, and one whose lovable disposition is revealed by her diary. "She stood high in the opinion of her contemporaries as a woman of culture and charm, whose memory is still green in the family at the present day."

It was into the great manor house at Kalbe, a rambling, spacious abode of one storey only, that Frau von Alsvenleben went as a bride in the summer of 1793, and never has a sweeter record of the home life of two devoted people been written than that of Werner and Annaliebe von Alsvenleben.

"High thinking and gentle manners had ever been the rule in the dear old house, but the style of living was of the plainest. . . . There was no question of a retinue of servants. An old coachman (Johann), who had been twenty-five years in the family, and a few maids formed the entire establishment." "And yet how charming and home-like the old house must have been, with its wide hall, the walls hung with portraits of the ancestors, some of them in knightly armour, with great plumes in their steel helmets, and the ladies with white veils on their almost invariable golden locks." The first entry in the diary is made on the evening of her arrival in the new home. "O, dear and beautiful house, from henceforth to be my cherished home! My whole heart goes out to thee in greeting. . . . How I love thee, dear old house, with all thy countless nooks and crannies, out of which I seem to see the kindly little brownies creep who have worked so blithely for the long line of my dear Werner's forefathers. Oh, receive me too, the stranger, as a familiar friend!" A month later the serenity of the household was broken by a letter from a cousin requesting sanctuary for a young French lady. The writer, an officer in the German army, said his landlady had come to him in great anxiety about a young lady who had come under the escort of a friend from France, and had been confided to her care by him. She appeared to be suffering, not only physically, but mentally, from severe shock. She, Baroness Cecile, is described as "a lovely, distressful creature with great dark eyes," who had been *dame d'atour* to the hapless Princess de Lamballe, and only escaped from the guillotine by a miracle herself. On her way there she was rescued by her fiancé, Vicomte de Trellissak, who lost his life in contriving her escape." To the peaceful home at Kalbe the thoughts of the good Major instinctively turned; here the poor stricken fugitive would find a safe asylum. And here, indeed, she found all that was to be desired in loving sympathy, and tender ministrations at the hands of Frau von Alvensleben, during an attack of brain fever, which followed shortly after her arrival. With these kind friends she remained till the autumn of 1801, when she returned to Paris to find Napoleon installed as First Consul.

* Memoirs of the Baroness Cecile de Courtot. From the German by Jessie Haynes. (Heinemann. One vol.)

An extract from Frau von Alvensleben's diary, written after the departure of her guest, proves that time had but confirmed and deepened the feeling of affection with which, from the first moment of meeting, the unfortunate young baroness had inspired her—a feeling entirely reciprocal, as Cecile's letters breathe in every line loving gratitude for the kind hospitality received at the hands of her generous hostess. The entry in the diary referred to is as follows: "November, 1801. I have allowed some days to pass before allowing my grief to find vent in my diary; I was almost afraid to put my feelings into words. My tears have not ceased to flow. . . . Dear sweet soul! I miss my friend at every turn! On my work table I found this delicate little bouquet—a last sweet souvenir from my Cecile. . . . A posy of forget-me-nots and red roses surrounded by a wreath of immortelles, all exquisitely painted in water colours. Written underneath are the words—

'Aime que j'aime,
Tu aimeras toi même.'

I have fixed it here to mark the boundary across which a new life must begin for the bereft of my soul's companion. How we all loved her!" I have dwelt on the more personal side of the Baroness's memoirs because many of the historical incidents are well-known already, and gain interest in perusal merely, from being related by an eye witness.

Upon her return to Paris she, through the good offices of Josephine, obtained an audience of Bonaparte with the object of gaining the restitution of her family estates. The meeting is dramatically described. "Letting his large, clear eyes rest on me for a moment with a piercing gaze, he said brusquely, 'Eh bien! what have you come about?' 'The restitution of my family property.' 'Of a truth, madame, I cannot complain that you are too prolix, but'—and he raised his voice to an angry pitch—'Why are you staring at me so strangely? I would have you remember, madame, that I am the head of the State, and as such require to be treated with proper respect.'" But his tone of angry remonstrance had no longer the terrors which the thought of the audience had at first inspired the fair auditor, for after looking at him for a moment, the sound of his voice with its peculiar accent, the imperious movement of the head, the cold, stern face with its clear cut pale features, recalled a scene far back in her life when she, a visitor near Brienne, had been saved from an infuriated bull by a young military student, and later, when the prizes were presented to the cadets, had had the good fortune to present a wreath of laurel leaves to her preserver —Napoleon Bonaparte—before whom she was now standing as a suppliant. "'So you were that sweet, kind girl, mademoiselle? Oh, ask me what you will, I promise beforehand to grant it, no matter what it is.' . . . 'Oh, *Sire*, what have I done to deserve this gratitude?' 'The royal title—for the first time—from your lips little prophetess . . . you set that laurel wreath on my young head in the far-off days . . . you whispered then "May it bring you luck." . . . I am a fatalist, mademoiselle . . . and since *you* have foretold it me, I *feel* the crown of France upon my brow, I see the sceptre of the great realm in my hand, and,' he concluded sadly, 'I have so few people about me in whom I can place real confidence, and stand so lonely here upon the heights, that it is an unspeakable relief to be able to unburden myself to a friend of my far-off youth.' The Baroness received the immediate restitution of her estates." These memoirs, full of personal interest, read like an almost impossible romance. The triumph of love over all obstacles, even death itself, and the reunion of the Baroness to her lover when all hope of meeting in this world had passed away, reads like the ending of a romantic fiction rather than the happy reality it was.

Much praise is due to the translator for her very admirable rendering of reminiscences full of romantic interest, through whose tragic scenes the record of devoted, unselfish lives, runs like a golden thread from end to end. A most charming and interesting work.

### Everybody's Opinion.

[Correspondence on all subjects is invited, but we cannot in any way be responsible for the opinions expressed by our correspondents. No communication can be entertained if the name and address of the correspondent is not given, as a guarantee of good faith but not necessarily for publication, or unless one side of the paper only is written on.]

### TYPHOID TWICE.

"DISTRICT NURSE" writes: In answer to "Nurse Helen," I beg to say it is possible to have typhoid more than once. I have myself had it twice severely, with an interval of three years only between the attacks.

### NURSING APPLIANCES.

"DISTRICT NURSE" writes: In your issue of the 26th ult. attention is drawn to an exhibit of nursing appliances. Being a Queen's Nurse I am familiar with the "District Bag," but I was much interested to hear of an "Operation Basket," also of the "Ward Box" referred to, and the baskets and cases for district nurses. I should feel indebted to anyone who could give me a little more information respecting these things, *i.e.*, the contents and probable cost of the "Operation Basket" and "Ward Box"; and the adaptability of the baskets or cases as substitutes for the somewhat cumbersome bag. I have a district in which at times a fair amount of surgery is done, there being no local hospital; hence my wish to know something further of the articles named.

### A VICTIM OF MAFEKING CELEBRATIONS.

"L. A. W." writes: In a remote village on the border of B—shire I am now nursing a victim of Mafeking celebrations. My patient, a man aged 28, and a blacksmith by trade, determined to show his patriotism by making a good noise, to effect which he charged three anvils with gunpowder, intending to fire them off simultaneously, and "wake the echoes far and near." But whilst boring a hole through one of the plugs the charge exploded, inflicting terrible injuries to his head and right hand. Just above the right eye a large wound was made, and a big piece of bone was blown away, exposing and injuring the brain, particles of the latter being found adhering to his cap. The hand is badly burnt, and there is a compound fracture of the forefinger, also a bad wound on the arm. The patient never became actually unconscious, and up to the present he is doing remarkably well.

### THE IDEAL MATRON.

"A MERE MAN" writes: I was much interested in reading "Some of the Difficulties of a Matron's Life" in your last issue. Your correspondent certainly succeeds in showing from the inside point of view that the necessary qualifications for the office of matron are not to be found in the average woman. For instance, a matron requires, according to your contributor, the "temper of an archangel." She must also be prepared not only to reprove judiciously, and to criticise circumspectly, but also to partake of breakfast while she is listening to the report of the night superintendent, and tea to a running accompaniment of taps at the door. She must be able to carve and to read prayers, she must have a smile perpetually ready for members of the visiting committee who wish to go round the wards. She must practically be always on duty, and she must never fail in temper, tact, nor judgment. It is not surprising that, in all the circumstances, your contributor arrives at the conclusion that the all-round ideal matron has yet to be evolved. As a mere man I am inclined to despair of her evolution.

### "OUR DUTY TO THE DYING."

"A PATIENT" writes: It is in beautiful words that "A Nurse" has discoursed on that problem of problems, whether the dying should, or should not, be left in ignorance of their critical state and the coming change. Surely, in all cases where it is in the least degree possible let us be told, so that we may spend our last hours, weeks, or months in the most profitable way. And this, not only from the religious point of view, for which of us have not some little treasures to be left in loving hands when we have "crossed the bar?" and where is the sister or friend who would not value with threefold tenderness the memento chosen for herself personally by the hands now cold and still? Without doubt there are cases where this course is not possible, such as the patient who would take a morbid delight in making her own shroud, or the other who would "have his fling while he could" because his case was hopeless. But I do think the majority of patients are brave enough to hear the sad verdict with courage and self-control, many of them being much happier in the knowledge than they were in uncertainty. Then how many of us, especially we chronic invalids, are apt to get impatient with our pain and to vent our irritability on those around, who would give the whole world, were it theirs, to see us well again? But when we know our hopeless condition, how quickly comes the thought, "It may be for such a short time I shall see those dear eyes beaming with love and tenderness; I must answer them in the same way, despite my suffering." And again, with many of us it is only when we know ourselves to be drawing nearer the other world week by week that we feel that ineffable peace which "passeth understanding." It is as though one had climbed wearily, step by step, to the top of a high hill, but the summit once gained how beautiful "the beyond." And how triumphantly we look back to tell the still plodding travellers of the glories in store, and to urge them onward. Is there any nurse or doctor living who would deny us the comfort of telling our nearest and dearest about that wonderful, all-radiating sense of love and mercy, "which softeneth the mystery and the parting?"

### PROPOSED MEMORIAL TO THE LATE LADY SUPERINTENDENT AT CRUMPSALL INFIRMARY.

MAJOR R. F. BALLANTINE, of Crumpsall, Manchester, writes: In a recent issue of your paper a short paragraph appeared concerning the funeral of the late Miss Elizabeth G. Hanan, who for 18 years filled the important position of lady superintendent of nurses at the Manchester Workhouse Infirmary at Crumpsall, having also for some years previously served the Select Vestry of the parish of Liverpool in a similar capacity at their infirmary at Brownlow Hill. As master of the Manchester Workhouse I was associated with Miss Hanan during the whole of her period of service there, and knowing her self-sacrificing life, the intensity of her devotion to duty, and the high estimation in which she was held by the hundreds of nurses trained by her during her many years of service, I have been disappointed to find that so far little notice has been taken by the papers devoted to nursing affairs of such a noble life as that which has now been brought to a close. As one of the pioneers of nursing on the higher plane—I am speaking now of the nursing profession of 20 years ago—Miss Hanan consistently and earnestly advocated the placing of the trained nurse in her true position, foreseeing then very clearly what would ultimately come to pass. Knowing as I do the loving care and devotion which were shown by Miss Hanan to all under her supervision, patients and nurses alike, and the fact that to the many nurses coming to Crumpsall for training, from all parts of the Kingdom—and even from abroad—she stood in the position of a mother and guardian, a true and trusted counsellor and guide, I am not surprised to find that a feeling of gratitude and affectionate regard has been shown on the part of many of her old nurses, and a desire to give practical expression to the same by contributing towards a suitable memorial of her to be erected in the Bristol Cemetery, where she is interred. I therefore venture to ask you to insert this letter in your next issue, and to request that anyone desirous of contributing towards the object in view, will be so good as to forward their contributions to me, not later than the middle of August, when the same will be duly acknowledged. Further particulars as to the description of monument, design, &c., will be published in your valued paper. I may add that small contributions will be appreciated equally with larger ones, as it is desired that all who feel an interest in the proposal should have an opportunity of sharing in this labour of love.

### For Reading to the Sick.

"O LORD, how manifold are Thy works, in wisdom hast Thou made them all; the earth is full of Thy riches."—*Ps.* civ. 24.

How glorious were the nightingales last night,
'Neath the dim April, warm, half-moonlit sky!
As from wood choirs and temples of delight,
The dewy streamside grass, the black thorn nigh,
They poured their melody.

Indeed! I heard it not! I looked around,
And deemed that night and silence had their fill;
From forest, fallow, distant lane, no sound
Save the dull dronings of the water mill:
The nightingales were still.

O dull of ear to hear! but mark thou this:
My ears were sharpened by a bed of pain;
Thus, out of sorrow God works often bliss,
And that flits by, and this shall still remain:
The nightingales no strain!!!

But *sursum corda!* may it not be so
That those sweet strains on Jordan's further side,
Unheard by souls who only this world know,
May yet to them not wholly be denied
Who drink the cup of woe?

—*J. M. Neale.*

**Reading.**

From where I lie I cannot see much; but the bright sunshine reaches me, and I can just see the tops of trees, and I can hear more than I can see. Through the open window I hear sweet summer sounds—the song of birds, and the rustling of leaves, and the many other sounds of nature. And sweet scents come up to me; the smell of mown grass, and of shrubs and flowers.

These sights and sounds and scents soothe and please me. Though I cannot get up and go out and enjoy them as others do, yet I have much quiet enjoyment of them here; for they are not far off, and they all speak to me of God.

It is this that makes me love them so. All these works of God seem to praise Him, and all make me feel His presence. In the song of birds I hear the voice of Him who made them. The rustling of the leaves in the breeze speaks of the wondrous power that sets each leaf in motion, and reminds me of the breath of His spirit. All these various sounds and scents speak to my senses and my heart of the almighty and loving Creator. The summer air and open window would not bring me half the pleasure I feel if all did not speak to me of God. "All Thy works praise Thee, O Lord."

Father, deal with me as Thy child. Give me the comfort of Thy spirit; sanctify to me these pains, this weariness. O, my Creator and Preserver, my never-failing Friend, put forth Thy healing, soothing, restoring, strengthening power. —*Bourdillon.*

Flowers preach to us if we will hear.
The rose saith in the dewy morn,
I am most fair;
Yet all my loveliness is born
Upon a thorn.

The lilies say: Behold how we
Preach without words of purity.
The violets whisper from the shade
Which their own leaves have made:
Men scent our fragrance on the air,
Yet take no heed
Of humble lessons we would read.
But not alone the fairest flwers:
The merest grass
Along the roadside where we pass,
Lichen and moss and sturdy weed,
Tell of His Love Who sends the dew,
The rain and sunshine too,
To nourish flower and seed.—*Christina Rossetti.*

### Notes and Queries.

THE Editor is always willing to answer in this column, without any fee, all reasonable questions, as soon as possible.

But the following rules must be carefully observed:—

1. Every communication must be accompanied by the name and address of the writer.
2. The question must always bear upon nursing, directly or indirectly.

If an answer is required by letter a fee of half-a-crown must be enclosed with the note containing the inquiry.

*Nursing Institutions Abroad.*

(113) I should be much obliged if you would kindly give me the names and addresses of nursing institutions in Rome, Florence, and Vienna.—*St. Anthony.*

You had better write to the English Consuls or chaplains at the places named for particulars.

*Dentistry.*

(114) Would you kindly tell me what is the highest wage of a mechanical dentist per week if taught in all its branches? I have apprenticed my son, and now find I am unable to have him taught the surgical work.—*Nurse Anxious.*

Good mechanical dentists receive high pay, from £3 to £5 per week.

*Religious Melancholy.*

(115) Could you kindly tell me of any home for nervous patients where a lady could be received who is suffering from religious depression? It must be comfortable, but the terms not more than 15s. per week.—*B. W.*

You will find a list of private asylums and licensed houses for the mentally deranged in "Hospitals and Charities," published by the Scientific Press.

*Carrying Baby.*

(116) "Nemo," as a thoroughly trained maternity nurse, takes her baby out (weather permitting) frequently during the month. Is this an extraordinary thing? as some very unpleasant remarks have been passed, to the effect that no trained nurse lowers herself by carrying a baby out.

Untoward remark upon such an obvious duty can only have been prompted by jealousy.

*Short Training.*

(117) "H. M. S." would be glad to know if the editor could recommend to her an institution or hospital (in or near London preferred) where she could receive, in less than three years, a training sufficient to qualify her to take a post as nurse-attendant to an invalid lady or gentleman.

The matrons of many small hospitals give short periods of training which would qualify a suitable person as nurse attendant. "H. M. S.' must, however, be careful to select a well-managed institution, as so much of the training depends upon the matron herself in a small place.

*Provident Dispensary.*

(118) Would you kindly inform me the best way of starting a provident dispensary in a London suburb? I should be very grateful for full particulars.—*Nemo.*

Provident dispensaries should always be arranged by the medical staff by whom they are to be run, or should be under the guarantee of some public body. In London the proper body to apply to is the Metropolitan Provident Medical Association, 5, Lamb's Conduit Street, W.C.

*Lunacy.*

(119) A question is put to us by Nurse Ferguson as to the action of one of the Commissioners in Lunacy. We cannot, however, discuss such a subject upon an *ex parte* statement, especially in view of the family complications it displays. The matter seems to have been before the Master in Lunacy, who has evidently been quite alive to the point raised by Nurse Ferguson, so that there is every reason to suppose that the case has been properly determined.

*Nauheim.*

(120) "Alison" very kindly sends the following information in reply to a recent inquiry about Nauheim: "There is an Englsih pension (kept by an English lady) just opened here; it is near the station and the baths, and is beautifully clean. The terms are from eight marks a day for each person; this includes everything but lights or fire in bedroom. There are also innumerable hotels and villas, but very few are really cheap."

*Return for Service.*

(121) Would you kindly tell me of an institution where a trained nurse would learn midwifery in return for services?—*Nurse Sara.*

It is not easy to secure training on the terms you name. It is better, and also cheaper in the long run, to enter a lying-in hospital and pay the very moderate fees charged.

**Standard Books of Reference.**

"The Nursing Profession: How and Where to Train." 2s. net.
"The Nurses' Dictionary of Medical Terms." 2s.
"Burdett's Series of Nursing Text-Books." 1s. each.
"A Handbook for Nurses." (Illustrated.) 5s.
"Nursing: Its Theory and Practice." New Edition. 3s. 6d.
"Helps in Sickness and to Health." Fifteenth Thousand. 5s.

All these are published by THE SCIENTIFIC PRESS, Ltd., and may be obtained through any bookseller or direct from the publishers, 28 & 29, Southampton Street, London, W.C.

